# A grid-based sample design framework for household surveys

**DOI:** 10.12688/gatesopenres.13107.1

**Published:** 2020-01-27

**Authors:** Gianluca Boo, Edith Darin, Dana R. Thomson, Andrew J. Tatem

**Affiliations:** 1 WorldPop, School of Geography and Environmental Science, University of Southampton, Southampton, SO17 1BJ, UK; 2 Department of Social Statistics and Demography, University of Southampton, Southampton, SO17 1BJ, UK; 3 Flowminder Foundation, Stockholm, 11355, Sweden

**Keywords:** Demography, Household Surveys, Sample Design, Spatial Sampling, Gridded Population, Democratic Republic of the Congo

## Abstract

Traditional sample designs for household surveys are contingent upon the availability of a representative primary sampling frame. This is defined using enumeration units and population counts retrieved from decennial national censuses that can become rapidly inaccurate in highly dynamic demographic settings. To tackle the need for representative sampling frames, we propose an original grid-based sample design framework introducing essential concepts of spatial sampling in household surveys. In this framework, the sampling frame is defined based on gridded population estimates and formalized as a bi-dimensional random field, characterized by spatial trends, spatial autocorrelation, and stratification. The sampling design reflects the characteristics of the random field by combining contextual stratification and proportional to population size sampling. A nonparametric estimator is applied to evaluate the sampling design and inform sample size estimation. We demonstrate an application of the proposed framework through a case study developed in two provinces located in the western part of the Democratic Republic of the Congo. We define a sampling frame consisting of settled cells with associated population estimates. We then perform a contextual stratification by applying a principal component analysis (PCA) and *k*-means clustering to a set of gridded geospatial covariates, and sample settled cells proportionally to population size. Lastly, we evaluate the sampling design by contrasting the empirical cumulative distribution function for the entire population of interest and its weighted counterpart across different sample sizes and identify an adequate sample size using the Kolmogorov-Smirnov distance between the two functions. The results of the case study underscore the strengths and limitations of the proposed grid-based sample design framework and foster further research into the application of spatial sampling concepts in household surveys.

## Introduction

Research and policymaking often require demographic data, such as population enumerations and age and sex structures. While these data have been historically derived from national censuses ^1^, the past 40 years have witnessed an increasing interest in the use of household surveys for demographic estimations ^2^. Starting from 2000, for instance, the US Census adopted the dual system estimation that complements the national census with a richer set of demographic and socio-economic characteristics captured using household surveys ^3^. This kind of survey provides a cost-effective way to access an extensive range of attributes that can be ultimately generalized to a larger population of interest ^4^. Generalization is especially valuable in low- and middle-income countries with outdated, inaccurate or incomplete censuses, where a sample of representative households can be used to estimate demographic data ^5^.

Traditional sample designs for household surveys build on three pillars — the sampling frame, sampling design, and estimator ^6^. The sampling frame consists of a list of all potential sampling units ^7^, the sample design defines the probability of any given unit to be sampled ^8^, and the estimator determines the rule to generalize the estimate (for example, recovering the mean characteristics of the population of interest using the mean characteristics of the sampled households) ^6^. In low- and middle-income countries, these sample designs are generally set up in two stages because of logistical and financial considerations ^9^. This form of multistage design involves the initial sampling from the primary frame, which is composed of non-overlapping enumeration units. Following the definition of a secondary frame resulting from the enumeration of all households in the sampled enumeration units, households are finally sampled ^9^.

The primary frame is an essential aspect of two-stage sampling designs because it is meant to provide an accurate, complete, and up-to-date representation of the distribution of the population of interest ^7^. This is defined using enumeration units and population counts retrieved from the most recent national census, an exercise that, in the best-case scenario, is carried out on a decadal basis ^10^. Census data become rapidly outdated because a maximum time-span of two years should typically occur between the definition of the sampling frame and the sample design implementation ^7^. As a consequence, sample designs for household surveys are increasingly relying on alternative sampling frames, typically derived from gridded population estimates ^10^. These estimates are produced through top-down spatial disaggregation of national census data ^11^ or bottom-up spatial interpolation based on household survey data collected within small geographic areas ^12^.

Adopting a gridded sampling frame requires adjusting the three pillars of household sample design conceived for one-dimensional listings to a bi-dimensional geographic space ^4^. This adjustment can be achieved by considering the three core concepts of spatial sampling — the random field, the design, and the estimator ^13^. The notion of random field formalizes the population of interest through a bi-dimensional random process characterized by errors, trends, autocorrelation, and stratification ^14^; the design reflects the specificities of the random field in the selection of sampling units; and the estimator defines the generalization of the estimate retrieved from the sampling units to the entire sampling frame ^15^. Despite the need for bridging sample designs for household surveys and spatial sampling, explicit joint methodological frameworks are currently still rare ^10^.

To fill this knowledge gap, we propose a grid-based sample design for household surveys that embeds the three core concepts of spatial sampling ^13^. In doing so, the gridded sampling frame is formalized as a bi-dimensional random field ^13^; the design considers spatial trends, spatial autocorrelation, and stratification through a contextually stratified ^16^ proportional to population size sampling ^5^; a nonparametric estimator is used to assess the sampling design and inform sample size estimation ^17^. We demonstrate the application of this sample design framework with a case study developed in two provinces located in the western part of the Democratic Republic of Congo. This country had its last census over 30 years ago, and sampling frames for household surveys are still based on these extremely outdated population figures ^18^. The results of the case study provide valuable insights into the implementation of the proposed framework and foster further research into grid-based sample designs.

## Methods

### The grid-based sample design framework

**Figure 1.**
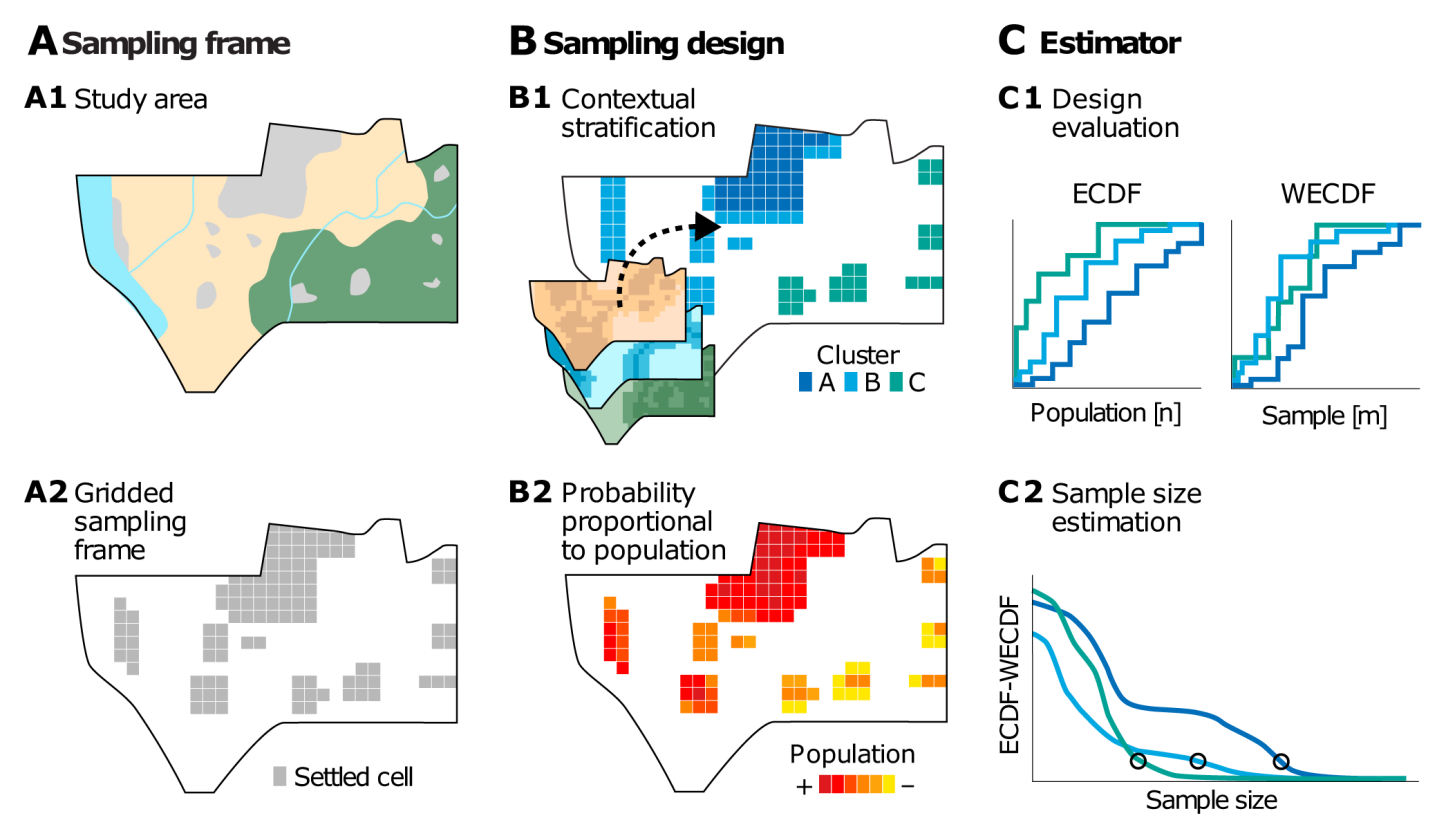
The grid-based sample design framework. The key elements of this framework are the sampling frame ( **A**) defined by deriving from the study area ( **A1**) the gridded sampling frame ( **A2**); the sampling design ( **B**) consisting of contextual stratification ( **B1**) and sampling proportional to population size ( **B2**); and the estimator ( **C**) where the empirical cumulative distribution function and the weighted empirical cumulative distribution function are used to evaluate the design ( **C1**) and estimate sample size ( **C2**).

Figure 1 shows the proposed grid-based sample design framework, which embeds the core concepts of spatial design into the three pillars of household sample design. First, the sampling frame ( Figure 1A) is formalized as a bi-dimensional random field, defined by superimposing a square grid to the study area, where the presence of settled area defines the sampling cells. The sampling design ( Figure 1B) reflects the characteristics of the random field, namely, spatial autocorrelation and spatial heterogeneity, by combining contextual stratification and proportional to population size sampling techniques. Lastly, an estimator ( Figure 1C) of nonparametric nature, namely the cumulative distribution function (CDF), is used to evaluate the sampling design and guide sample size estimation in a simulation study. The three elements of the proposed grid-based sample design framework are presented in detail in the next sections. The proposed grid-based sample design framework can be implemented using the R statistical language ^19^ in RStudio 3.5.2 ^20^, using the following packages — gridsample 0.2.1 ^21^, raster 3.0-7 ^22^, sf 0.8-0 ^23^, and spatstat 1.61-0 ^24^.

### Sampling frame

The notion of sampling frame is at the core of household sample design because it ensures that every household has a known probability of being surveyed ^7^. This concept, however, is not frequently adopted in other disciplines, such as environmental sciences, because full listings are considered impractical or even impossible ^13^. To overcome this issue, in the domain of geostatistics, the complete listing of the population of interest is replaced by the listing of the geographical location where it can be found ^16^. For this purpose, a regular geometric grid with square or hexagonal patterns is overlaid on the study area to enable equal sampling probability ^25^. Given the heterogeneous geographic distribution of the human population, in the past, the use of gridded sampling frames has been discouraged for household surveys ^16^. However, other spatially explicit sampling frames, for instance, based on parcel boundaries ^26^ or air pollution levels ^16^, have already been adopted in the past for household sampling.

Gridded population sampling frames are being increasingly adopted in household sampling carried out in low- and middle-income countries with outdated census frames ^10^. This is because, in some instances, traditional sampling frames lack complete geographic coverage, well-defined geographic boundaries and up-to-date population data ^9^. Conversely, a gridded sampling frame provides comprehensive coverage of well-defined regular sampling units — the grid cell ^5^. The increasing availability of high-resolution gridded population estimates, with cells measuring between 30 ^27^ and 250 meters ^28^, also enables deriving sampling frames of relatively fine spatial resolution. Whether gridded population estimates have known inaccuracies connected with the quality of the input datasets ^5^ and selected spatial disaggregation techniques ^11^, they are generally considered to provide a more accurate approximation of the geographical distribution of population counts than outdated census enumerations ^5, 11^.

While most gridded population estimates are constrained to settled areas ^11^, WorldPop top-down estimates provide a continuous population-count value across all land masses to ensure that sparsely-populated areas are not omitted ^29^. This dataset also offers the advantage of a systematic global coverage and an accuracy assessment ^29^. Furthermore, a gridded sampling frame derived from WorldPop top-down estimates can be refined using global settlement datasets such as the Global Urban Footprint (GUF) ^30^ and the Global Human Settlement Layer (GHSL) ^28^ using the settled area as a limiting ancillary variable ^31^. The sampling frame, defined based on the population counts within settled cells, can be formalized as a random field (ℜ), where the population count in a settled cell ( *X*) is distributed across a bi-dimensional parameter space (ℝ ^2^) as a function of its geographic coordinates ( *l*) ( Equation 1).

Equation 1 $\;ℜ=\left\{X(l),l\in {\mathbb{R}}^{2}\right\}$

The population count within a settled cell ( *X*( *l*)) is influenced by the following features. First, spatial autocorrelation, or first-order non-stationarity, since *X*( *l*) is expected to be similar when the settled cells are close to one another ^32^. This condition violates the underlying assumption of an independently distributed population governing probabilistic sampling and involves a loss of sampling efficiency ^33^. Second, spatial heterogeneity, or second-order non-stationarity, as *X*( *l*) is likely to differ across *l* in different geographic contexts, such as urban/rural or mountainous/flat areas ^34^. This situation also contravenes a crucial assumption of probabilistic sampling, namely, the presence of an identically distributed population ^35^. The third variable is discreteness, as *X*( *l*) is not continuous across all potential *l* but limited to settled areas only ^31^. This last characteristic implies that traditional spatial sampling techniques are not directly applicable because the sampling frame is not a continuous surface but constrained to settled cells only ^13^.

### Sampling design

Opposite to geostatistics, household surveys adopt design-based sampling strategies because the population of interest is considered unknown but fixed and entirely measurable ^4^. Within different design strategies, household surveys in low- and medium-income countries are often based on two-stage sampling designs ^9^. This design involves drawing enumeration units from a primary sampling frame with probability proportional to population size, in which a number of households are subsequently randomly surveyed ^7^. First-stage sampling is crucial to improve sampling efficiency because it can incorporate characteristics of the random field ^6^. For example, enumeration areas may be selected with probabilities proportional to their population sizes to better account for spatial heterogeneity and to include densely populated areas that would likely be excluded from a random sample. However, the scarce accuracy of the population enumerations retrieved from the last census and the definition of coarse strata can limit the efficiency of proportional to population size sampling for household surveys ^36^.

Stratified sampling assumes that the population of interest can be partitioned into more homogeneous subpopulations, or strata ^13^. This is to capture the spatial heterogeneity in the population of interest globally, and, consequently, to reduce the in-sample spatial autocorrelation ^6^. Stratification can be based on prior knowledge, pre-sampling, or proxy variables ^37^. In household sampling, strata often consist of a proxy reflecting the urban/rural divide ^8^, a strategy that is reproduced in existing grid-based sampling designs to provide independent estimates for planning and decision-making ^5^. The use of bi-dimensional gridded sampling frames enables finer contextual stratification by incorporating information on geographic phenomena influencing the distribution of the population of interest ^16^. This can be achieved by accessing ancillary gridded datasets related to socio-economic (e.g., distance to major roads and urban centres) or physical characteristics (e.g., terrain and climate) that are embedded in top-down population models ^38^.

For each ancillary dataset, the cell values intersecting the settled cells define a high-dimensional space describing geographical context. This approach enables to define contextual strata by combining two popular methods for dimensionality reduction ^39^ — principal component analysis (PCA) ^40^ and *k*-means classification ^41^. PCA is meant to reduce the number of correlated random variables into a set of linearly uncorrelated principal components ^42^. The number of principal components can be selected by assessing the proportion of the total variance explained, which should generally be above 80–90% ^43^. The principal components of the high-dimensional contextual space can be further reduced using a *k*-means classification ^39^. This method enables to capture intrinsic structures by minimizing heterogeneity within clusters and maximizing the heterogeneity across clusters based on the mean of the principal components. The number of clusters can be assessed using the “elbow” method applied to the variance explained (i.e., the within-cluster sum of squares) ^44^, but also by inspecting whether the spatial distribution of the resulting clusters produces meaningful contextual strata.

Within each stratum, proportional to population size sampling has a straight-forward implementation in gridded sampling designs, through dedicated software packages ^5^ and web platforms (e.g. https://gridsample.org). The crucial feature of proportional to population size sampling is the use of gridded population datasets. For this purpose, several top-down gridded population datasets are currently available globally (e.g., GHS-POP ^28^, GPWv4 ^45^, LandScan ^27^, and WorldPop ^29, 46^, while bottom-up datasets are only being produced in a limited number of countries ^12^. These datasets have different characteristics and fitness for use that should be carefully considered in the sampling design implementation ^11^. 

The probability scheme resulting from stratified proportional to population size sampling $\left(\pi_{i}^{\left(SPPS\right)}\right)$ can be summarized as the joint probability of stratified sampling $\left(\pi_{i}^{\left(S\right)}\right)$ and sampling proportional to population size $\left(\pi_{i}^{\left(PPS\right)}\right)$ ( Equation 2).

Equation 2 $\;\pi_{i}^{\left(SPPS\right)}=\pi_{i}^{\left(S\right)}\times \pi_{i}^{\left(PPS\right)}$

The probability of selecting a specific cell *X _i_* in the design $\pi_{i}^{\left(S\right)}$ is contingent on the size of the stratum it belongs to ( *S _i_*), where *n _S_* is the number of sampled settled cells in the stratum *S _i_* and *m _S_* the total number of settled cells in the stratum *S _i_* ( Equation 3).

Equation 3 $\;\pi_{i}^{\left(S\right)}=\frac{n_{S}}{m_{S}}$

The probability of selecting a specific cell *X _i_* in the design $\pi_{i}^{\left(PPS\right)}$ is relative to its population size and the total size of the population, in other words, the sum of the population counts for each cell $\sum_{l=1}^{n_{S}}X_{l}$ ( Equation 4).

Equation 4 $\;\pi_{i}^{\left(PPS\right)}=\frac{X_{i}}{\sum_{l\in {\mathbb{R}}^{2}}X_{l}}$

Based on the probability scheme specified above, it is possible to produce an unbiased estimator that can be used to evaluate the sampling design and inform sample size estimation.

### Estimator

In household sampling design, the estimand is a parameter summarizing the random variable of interest, such as the mean, variance, or total ^8^. Typical examples are the mean proportion of children under five years old or the number of women of child-bearing age. In this setting, the estimator is built using a parametric attribute of the random variable of interest ^47^. However, the use of nonparametric estimators enables to retrieve the characteristics of the entire random variable ^48, 49^. In the case of sample design for household surveys, the random variable consists of the population count across settled cells, where a large number of cells have medium-to-low population counts and only a few have high population counts. To capture the characteristics of the entire population of interest, the estimand becomes the full probability distribution of the random variable through its CDF ^50^. The CDF ( *F _X_*( *x*)) summarizes the probability for the population count within a settled cell ( *X _i_*) of being lower or equal to *x*. Given the law of large numbers, the CDF can be approximated using the empirical CDF (ECDF) (F^m(x)) for a number *m* of sampling frame cells ( Equation 5).

Equation 5 $\;{\hat{F}}_{m}(x)=\frac{1}{m}\sum_{i=1}^{m}I\left\{X_{i}\leq x\right\},\;\;\text{where}\;I\left\{X_{i}\leq x\right\}=\left\{ \begin{array}{l} 1\;if\;X_{i}\leq x\\ 0\;otherwise \end{array}\right.$

Given that the proposed sample design is not random but probabilistic, the estimator needs to be weighted for the respective probability scheme ^51^. Typical parametric estimators, such as the mean or total, can be weighted using the Horvitz-Thompson estimator, by implementing the inverse of the probability scheme ^47^. This concept can be extended to nonparametric estimators, by weighting the ECDF using the inverse of the probability scheme, and producing a weighted empirical cumulative distribution function (WECDF) $\left({\hat{G}}_{n}(x)\right)$ for the number of sampled cells ( *n*) ( Equation 6).

Equation 6 $\;{\hat{G}}_{n}(x)=\frac{1}{n}\sum_{i=1}^{n}W_{i}\;I\left\{X_{i}\leq x\right\},\;\text{where}\;W_{i}=1/\pi_{i}^{\left(SPPS\right)}$

In household surveys, the sample size is typically determined using a power analysis applied to the parametric estimator, which is assumed to be normally distributed for large sample sizes ^8^. For nonparametric estimators, such as the WECDF, a simulation study can enable evaluation of the sample size required to provide an accurate representation of the population of interest across the different strata ^17^. For this purpose, the same gridded population data used in proportional to population size sampling can serve as a proxy for the entire population of interest. The population counts across sampling frame cells are used to derive the ECDF for the entire population of interest and the WECDF for different sample sizes, and compare the two distributions using a nonparametric statistic — the Kolmogorov-Smirnov distance ( *D _m,n_*) ^52^ ( Equation 7).

Equation 7 $\;D_{m,n}={sup}_{x}|{\hat{F}}_{m}(x)-{\hat{G}}_{n}(x)|$

*D _m,n_* is based on the maximum distance between ${\hat{G}}_{m}(x)$ for the entire population of interest across *m* settled cells, and (F^n(x)) for the population within a varying number of sampled cells *n*. While *n* increases iteratively, it is possible to assess the associated changes in *D _m,n_*. However, given that *D _m,n_* is extremely sensitive to the shape of the two distributions, the process of sampling *n* settled cells should be replicated and averaged to provide a robust assessment of *D _m,n_*. The use of nonparametric estimators (i.e., the ECDF and the WECDF) and statistic (i.e., the Kolmogorov-Smirnov distance) typically requires large sample sizes to capture the entire range and variability of population counts within settled cells. This process can be optimized by estimating sample size for each stratum independently ^13^.

## Case study

We demonstrate an application of the proposed grid-based sample design framework in two provinces in the western part of the DRC. This country is the second-largest by area and the fourth-most-populous in Africa. However, official population figures are currently lacking because the last census was carried out over thirty years ago, in 1984. Attempts to produce demographic data are routinely being carried out using population estimates and projections (e.g., https://population.un.org/wpp), as well as national surveys ^18^. Six national surveys have been carried out in the DRC since 2004 — two Demographic and Health Surveys (DHS) in 2013–2014 and 2017–2018, a Multiple Indicator Cluster Survey (MICS) from UNICEF in 2010, two Enquête 1-2-3 Surveys from the Congolese National Statistics Office in 2005 and 2012, and a Comprehensive Food Security and Vulnerability Analysis (CFSVA) from the World Food Programme in 2011–2012. These surveys have been developed using outdated sampling frames based on the census data of 1984, which has been shown to introduce uncertainty in both the collected survey data and the derived demographic information ^18^.

### Study area

**Figure 2.**
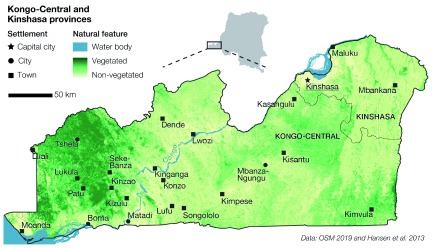
The study area comprising the Kongo-Central and Kinshasa provinces. Cities and towns develop mostly across the Congo river basin, while smaller towns can be found in the sparsely-populated plateau at the North-West and South-East of the study area. At elevated locations, the vegetation is prominent with the rain forest at the North-West and the savannah at the South-East.

The study area covers the Kongo-Central and Kinshasa provinces, in the Democratic Republic of the Congo. Together, these provinces constitute the most dynamic socio-economic region of the country. In this region, approximately 80% of the population lives in urban areas — in the capital city of Kinshasa, the cities of Boma and Matadi, and a number of smaller cities and towns ^53^. Figure 2 shows that urban areas develop from South-West to North-East, from the harbour town of Moanda, across the Congo river basin, to the vast agglomeration of the capital city Kinshasa. The remaining of the study area lies on a sparsely-populated plateau, where smaller towns (e.g., Kinganga and Mbankana) act as sub-regional centres for the surrounding villages and hamlets. In this sector, the vegetation is denser than in the Congo river basin, as the rain forest is prominent at the North-West and the savannah at the South-East. These particular urbanization patterns, and the consequent geographic distribution of population, are connected with the diverse socio-economic, infrastructural, environmental, physical, and climatic characteristics of the study area ^53^.

### Gridded sampling frame

**Figure 3.**
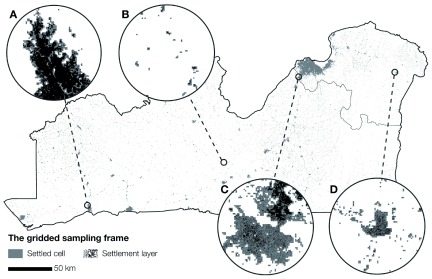
The settled cells constituting the gridded sampling frame. The gaps between settlement layer and the settled cells tend to vary considerably across the urban area of Boma (A), the suburban areas at the outskirts of Kinshasa (C), the town of Mbankana (D), and the rural area north of the town of Kimpese (B).

We accessed a settlement layer produced by the Oak Ridge National Laboratory using feature extraction from high-resolution imagery for population modelling work undertaken in the Kinshasa and Kongo-Central provinces. The settlement layer consists of settlement polygons of approximately 7 meters resolution that were subsequently subset to the official province boundaries provided by the Central Bureau of Census (BCR) of the Democratic Republic of the Congo. Comprehensive metadata are provided in Table 1. The polygons were rasterized based on a reference grid with a resolution of 3 arc-seconds, approximately 90 meters. The presence of at least one settlement polygon designated a settled cell — a gridded sampling unit. Figure 3 shows the gridded sampling frame, which comprises 211,831 settled cells. A large number of settled cells can be observed in the cities of Kinshasa, Boma, and Matadi, while more scattered settlement patterns can be observed in the rest of the study area. In more urbanized areas, such as in the city of Boma ( Figure 3A), the settled cells tend to match the extent of the settlement layer. Conversely, in suburban areas ( Figure 3C), towns ( Figure 3D), and rural areas ( Figure 3B) the gaps between the settlement layer and the settled cells become larger because the built-up area is more scattered.

**Table 1.** Metadata for the datasets used in the case study. The column “Type” indicates the characteristics addressed. The column “Format” describes the type of input data. The column “Type” defines the type of variable. The column “Source” reports the links to the datasets used in the case study.

Type	Name	Provider	Year	Format	Variable	Source
SE	Distance to conflict points	Armed Conflict Location and Event Data (ACLED) Project	2016	VECT	CONT	https://www.acleddata.com/data/
SE	Travel distance to cities	Malaria Atlas Project (MAP)	2015	RAST	CONT	http://doi.org/10.1038/nature25181
INF	Distance to major roads	OSM/WorldPop	2016	RAST	CONT	https://www.worldpop.org/doi/10.5258/SOTON/WP00644
INF	Light intensity at night	VIIRS/WorldPop	2016	RAST	CONT	https://www.worldpop.org/doi/10.5258/SOTON/WP00644
ENV	Degree of urbanization	GHS-SMOD	2015	RAST	CAT	https://ghsl.jrc.ec.europa.eu/ucdb2018visual.php
ENV	Land cover	ESA-CCI	2015	RAST	CAT	https://www.esa-landcover-cci.org
PHY	Elevation	SRTM/WorldPop	2000	RAST	CONT	https://www.worldpop.org/doi/10.5258/SOTON/WP00644
PHY	Slope	SRTM/WorldPop	2000	RAST	CONT	https://www.worldpop.org/doi/10.5258/SOTON/WP00644
CLIM	Rainfall	WorldClim	1960–2000	RAST	CONT	http://worldclim.org/version2
CLIM	Temperature	WorldClim	1960–2000	RAST	CONT	http://worldclim.org/version2
—	Population counts	WorldPop	2016	RAST	CONT	https://www.worldpop.org/doi/10.5258/SOTON/WP00645
—	Settlement layer	ORNL/WorldPop	2016	VECT	CAT	https://doi.org/10.5281/zenodo.3562191
—	Administrative boundaries	Central Bureau of the Census (BCR)	2018	VECT	CAT	*

*Datasets not publicly available.

SE, socio-economic; INF, infrastructural; ENV, environmental; PHY, physical; CLIM, climatic; VECT, vector; RAST, raster; CONT, continuous; CAT, categorical.

### Contextual stratification

**Figure 4.**
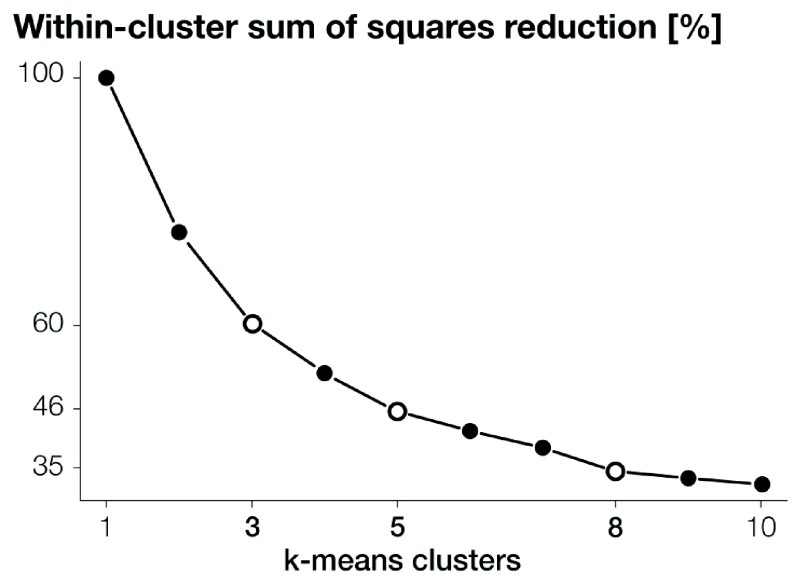
Within-cluster sum of squares reduction for *k*-means clusters spanning between one and ten. Three, five, and eight clusters are the best scenarios, according to the “elbow” method, for capturing the variance in the nine principal components derived from the gridded data attributes.

We retrieved ten gridded datasets describing the socio-economic (i.e., distance to conflict points and light intensity at night), infrastructure (i.e., distance to major roads and travel distance to cities), environmental (i.e., land cover and degree of urbanization), physical (i.e., elevation and slope), and climatic (i.e., temperature and rainfall) characteristics of the study area. These datasets have been selected because they represent key geospatial covariates in top-down population models developed by WorldPop ^38^. Comprehensive metadata are provided in Table 1. Gridded dataset attributes were extracted for the cells intersecting the settled cells, and categorical variables were “dummified”. A PCA was performed on the resulting 16 gridded data attributes and produced nine principal components that, together, explain 91.36% of the original variance. The nine principal components were then fed into a *k*-means clustering algorithm. Figure 4 shows the within-cluster sum of squares reduction for clusters spanning between one and ten. The “elbow” method suggests that three, five and eight clusters, with respectively 60.30%, 46.15% and 35.48% of the principal components’ variance explained, provide the best scenarios for capturing the variance in the principal components.

**Figure 5.**
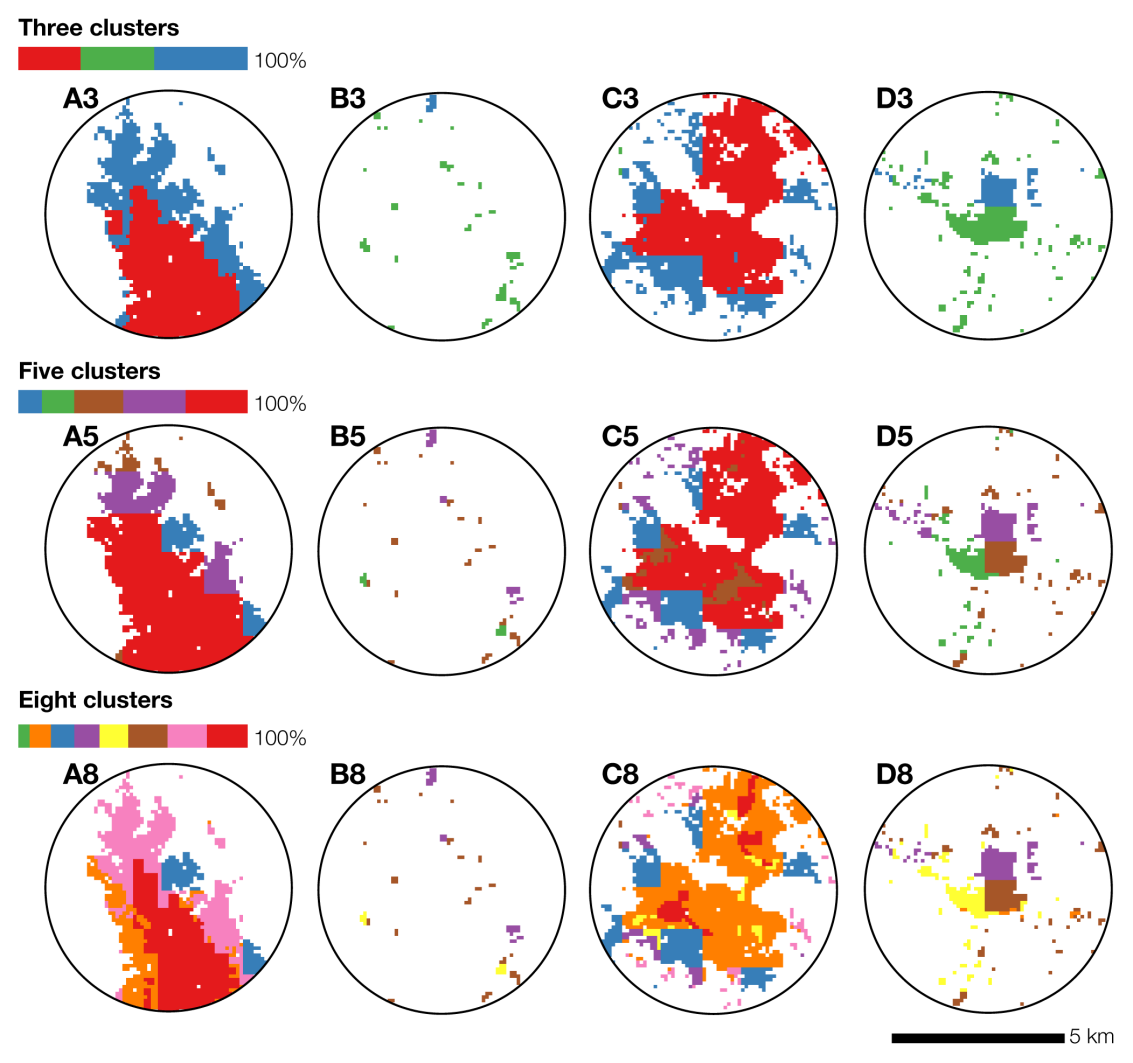
The spatial distribution of three, five and eight clusters for selected locations. The legends show the ratio of settled cells allocated to the different clusters. Overall, the spatial patterns resulting from the three scenarios produce comparable outputs, with a clear distinction between the urban (Boma — A) and suburban (outskirts of Kinshasa — C) areas versus the town (Mbankana — D) and rural area (North of Kimpese — B).

Figure 5 contrasts the spatial distribution of three, five and eight clusters across the urban area ( Figure 5A), suburban area ( Figure 5C), town ( Figure 5D), and rural area ( Figure 5B) presented in Figure 3. The legends show the ratio of settled cells allocated to the different clusters. Overall, the three scenarios produce comparable results, with a clear distinction between urban and suburban areas versus towns and rural areas. However, within urban and suburban areas, five and eight clusters seem to produce less realistic geographic patterns, with improbably sharp cluster boundaries ( Figure 5A5) and prominent “salt and pepper” effects ( Figure 5C8). Some of these patterns persist across the three scenarios, for instance, the sharp cluster boundaries occurring in the suburban area ( Figure 5C) and town ( Figure 5D). Within the three scenarios, the three-cluster scenario appears to produce the most realistic contextual strata. These contextual strata appear to reflect high (in red), medium (in blue), and low (in green) urban status.

### Probability proportional to population size

**Figure 6.**
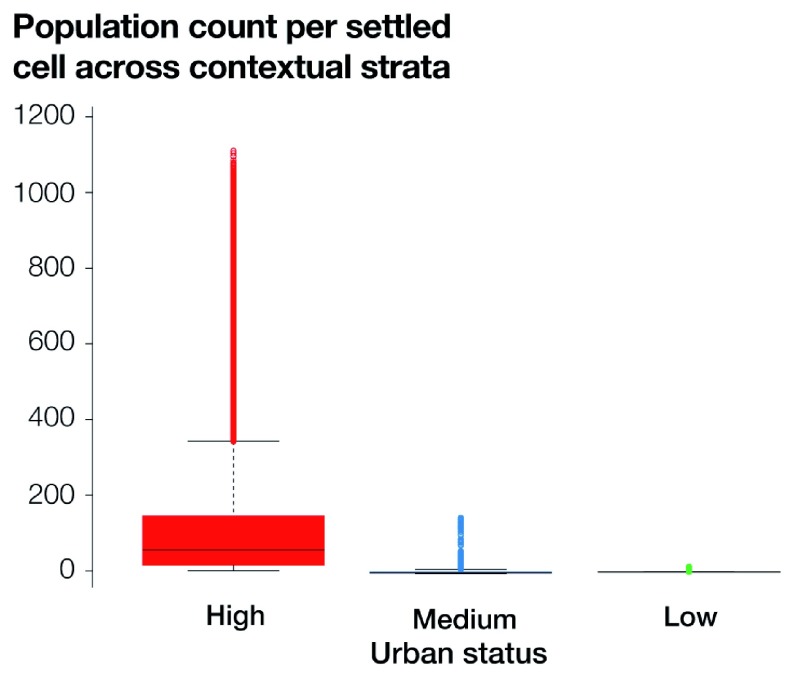
Distribution of population counts per sampling-frame cell across the contextual strata defined based on the three clusters scenario. The large horizontal black lines show the median, the boxes the interquartile range, the whiskers the minimum and maximum, and the dots the outliers.

We accessed high-resolution gridded population estimates for 2016 from WorldPop and allocated population figures to the corresponding settled cells. Comprehensive metadata are provided in Table 1. Figure 6 shows the distribution of the population counts per settled cell across the contextual strata derived from the three clusters scenario. Contextual strata labelled as high, medium, and low urban status include 26.91%, 40.14%, and 32.95% of the settled cells, respectively. Overall, the distribution of population counts per settled cell varies considerably across the three contextual strata, and this is consistent with the allocated labels of high, medium, and low urban status. The stratum characterized by high urban status has the highest median population count per cell of 55.58 and the largest outliers, with a maximum of 1109.41. Conversely, the stratum characterized by low urban status shows a very low median population count per cell of 0.15, with a maximum value of 13.97. The stratum with medium urban status also has a low median population count per cell of 1.39, but outliers are relatively important, with a maximum value of 146.74.

### Sampling evaluation

**Figure 7.**
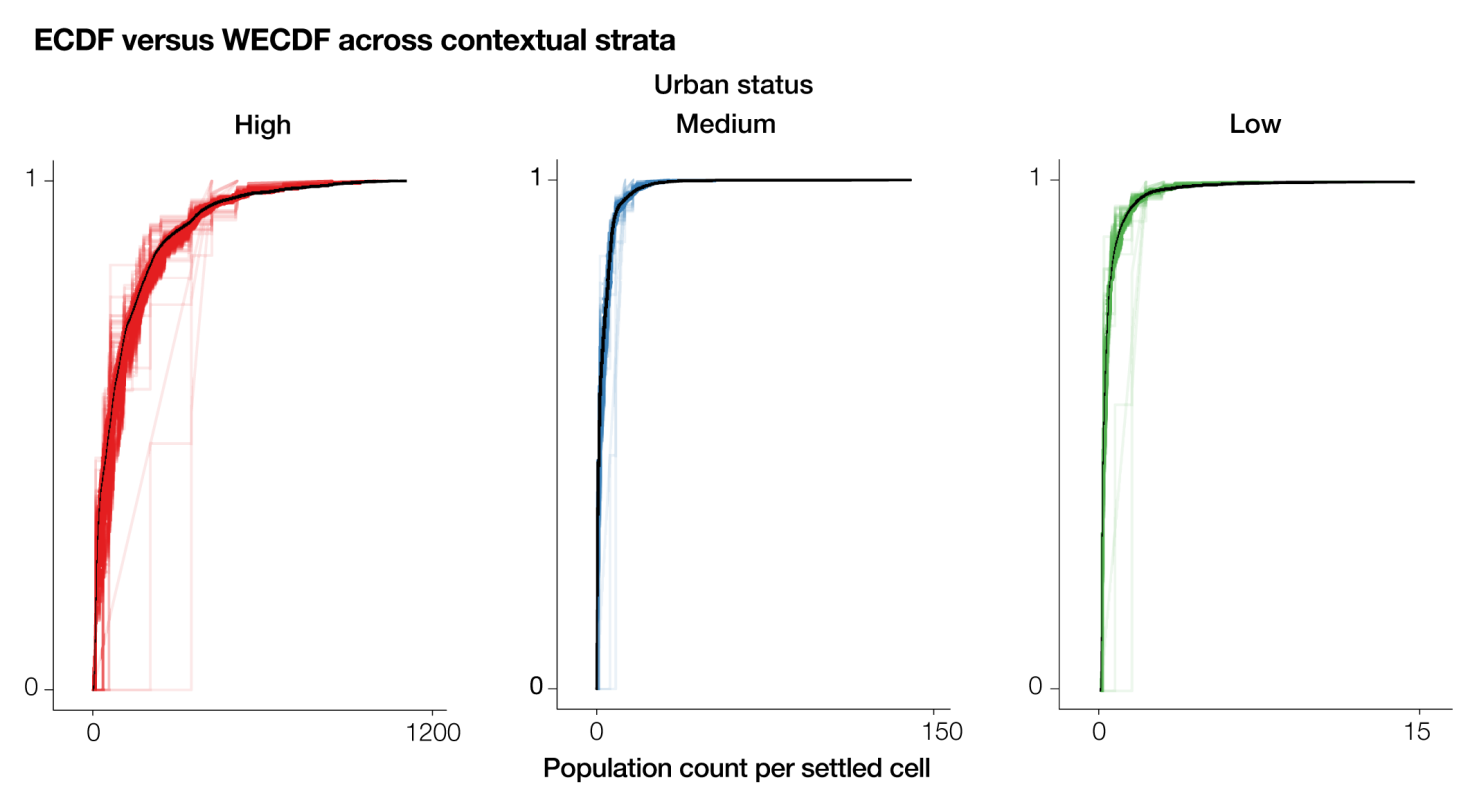
Empirical cumulative distribution function (ECDF) and weighted ECDF (WECDF). The ECDFs are depicted as black lines and the ECDFs as coloured lines. Sample sizes for the ECDFs span between 1 and 1000. The settled cells are selected using proportional to population size sampling for each contextual stratum (high, medium, and low urban status), independently.

We sampled settled cells from each contextual stratum proportionally to the respective population counts. Figure 7 contrasts the ECDF (black lines) to the WECDF (coloured lines). For each stratum, the ECDF lines depict the cumulative distribution of the population counts across all the settled cells, while the WECDF lines show the cumulative distributions of the population counts for a number of sampled grid cells spanning between 1 and 1000. Overall, the WECDF lines become less dispersed towards higher values and are mostly above the ECDF lines. Conversely, the WECDF lines tend to be more scattered for low-to-medium values and are mostly located below the ECDF lines. These results reflect the oversampling of settled cells with the highest population counts resulting from the proportional to population size sampling strategy. This expected pattern is predominant in the stratum characterized by high urban status, while it appears to be negligible in the strata with medium and low urban status.

### Sample size estimation

**Figure 8.**
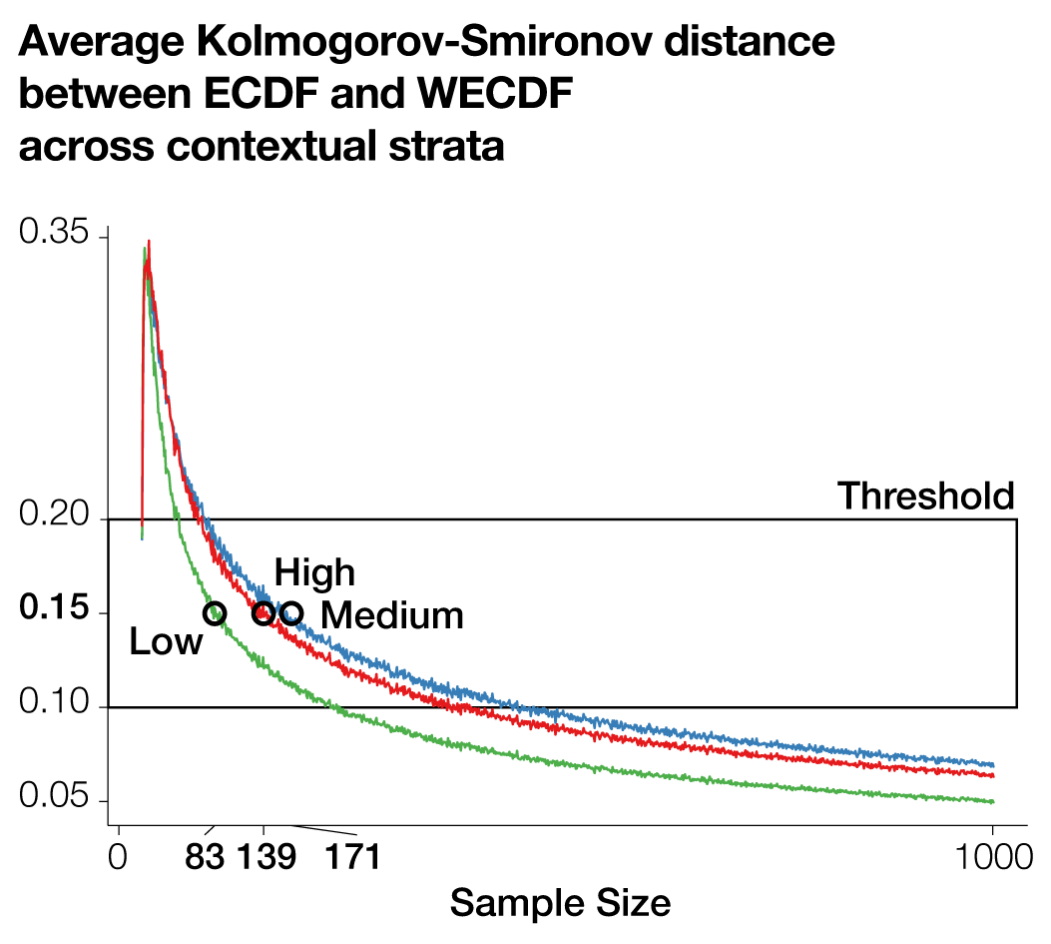
Average Kolmogorov-Smirnov distance for each contextual stratum. For sample sizes spanning between 1 and 1000, 1000 repetitions have been carried out and then averaged to produce a more robust assessment. The box highlights sample sizes resulting in reasonable distance metrics. The circles show the sample sizes resulting in a distance of 0.15.

We computed the Kolmogorov-Smirnov distance between the baseline ECDF and the WECDF for sample sizes spanning between 1 and 1000 across the different strata. We replicated this procedure 1000 times for the different sample sizes and averaged the distance metrics to provide a robust assessment of the distance between the two functions. Figure 8 shows the mean Kolmogorov-Smirnov distance for sample sizes spanning between 1 and 1000 across the different contextual strata. Overall, average distances show similar patterns across different strata. Low average distances can be observed for extremely low sample sizes that then spike before gradually decreasing as a function of sample size. This suggests that after discarding very low sample sizes — poorly recovering the reference population — and very high sample sizes — providing negligible improvements — it is difficult to estimate ideal sample sizes. However, Figure 8 suggests that a sample size threshold can be defined based on sensible distance values (e.g., between 0.10 and 0.20), and sample size can be allocated across strata to provide similar sampling performances. Figure 8 shows that, in order to achieve a sampling performance of 0.15, 139 settled cells should be sampled from the stratum with high urban status, 171 from the stratum with medium urban status and 83 the stratum with low urban status — 0.25%, 0.20%, and 0.12% of the respective settled cells.

### Sampled locations

**Figure 9.**
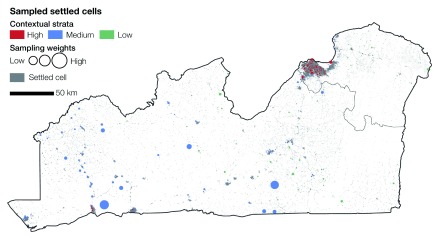
Sampled settled cells across the different contextual strata. The resulting sampling weights vary considerably across strata. Higher weights can be observed in areas of lower population counts per settled cell within the medium urban status stratum, while lower weights can be found in the sparsely populated low urban status stratum.

To obtain similar sampling performances, we sampled 139, 171 and 83 settled cells from the strata with high, medium, and low urban status, respectively, proportionally to population size. Figure 9 shows the sampled locations across the three strata and the sampling weights to be embedded in the estimator. The highest weights can be observed for the stratum of medium urban status, mostly across sparsely populated areas. Higher weights are also present in the stratum with high urban status, especially at the outskirts of Kinshasa. In this sector, the urban transition results in substantially lower population counts per settled cell, compared with the settled cells within the same stratum. The lowest weights can be observed across the strata with low urban status because its total population is by far the lowest.

## Discussion and conclusions

### Limits of traditional sample designs

In low- and middle-income countries, sample designs for household surveys are traditionally set up in two stages for logistical and financial considerations ^9^. This form of multistage sampling involves an initial sampling from the primary frame, which consists of non-overlapping enumeration units defined proportionally to population size ^7^. These enumeration units are typically derived from the last national census, which is usually carried out on a decadal basis ^54^. In reality, the time-spans between censuses can be even larger as, according to the United Nations’ Department of Economic and Social Affairs, 23 countries had the last census over ten years ago. Even when collected regularly, census data become rapidly outdated because a maximum time-span of two years should typically occur between the definition of the sampling frame and the household survey sampling and implementation ^7^. For this reason, traditional sample designs for household surveys are to be considered representative only at sporadic frequencies and for relatively short periods.

The uncertainty associated with non-representative sampling frames propagates through the sampling design to the estimator ^8^. As a consequence, the resulting household surveys can limit the accuracy of the derived demographic data ^18^. To tackle this issue, research in the domain of household sample design recently started to focus on the use of gridded population data to produce actionable sampling frames ^10^. Given the geographically explicit nature of gridded sampling frames, sample designs for household surveys can arguably benefit from spatial sampling techniques traditionally applied in natural sciences ^13^. To date, only a limited number of sample designs for household surveys have explicitly considered concepts of spatial sampling through the concepts of random field, sampling design and estimator. Two such studies reflect the characteristics of the random field in sample design using parcel boundaries ^26^ and air pollution levels ^16^. However, none of these studies explicitly considered the geographic distribution of the reference population in their sample design.

### Adopting gridded sampling frames

To tackle the limits of traditional sample designs, we proposed an innovative grid-based sample design framework for household surveys. This framework is centred around the concept of gridded sampling frame, a concept that is traditionally being adopted in natural sciences ^8^ and, more recently, in sampling for household surveys ^10^. The use of geographically explicit sampling units enabled us to revise the three pillars of traditional sample design — sampling frame, sampling design, and estimator — through the elements of the core components of spatial sampling ^13^. A key element of the proposed framework is formalizing the population distribution as a random field, and tackle spatial trends, spatial autocorrelation, and stratification of the reference population. These considerations are embedded in the sampling design, where contextual stratification ^8^ and population-weighted sampling ^36^ are used jointly to improve sampling efficiency. Both the sampling design and the sample size are assessed based on a nonparametric estimator to assess generalization to the entire reference population ^48, 49^.

We demonstrated an application of our proposed sample design framework with a case study developed in two provinces in the western part of DRC. In this country, existing sampling frames are typically developed based on outdated census figures dating from 1984. As a result, much demographic information produced through the six national surveys carried out since 2004 is highly uncertain ^18^. We built a gridded sampling frame for the study area consisting of settled cells of approximatively 90 meters spatial resolution. We then defined the two essential elements of our sampling design, namely the contextual strata based on a combination of PCA and *k*-means algorithm and the probability proportional to population size per settled cell retrieved from recent gridded population estimates. While the estimates are arguably uncertain because based on projections from the last national census, their geographic distribution is a reasonable approximation of the geographic distribution of population across the study area ^5, 11^. We assessed the sampling design by contrasting the ECDF for the population to the WECDFs for different sample sizes across the contextual strata. We also examined how sample size impacts the recovering the characteristics of the entire reference population across the different contextual strata. Lastly, we document and describe the geographic distribution of the sampled cells and the relative sampling weights to be embedded in the estimator.

### Challenges and next steps

The case study underscores some challenges of the proposed grid-based sample design. First, the spatial accuracy of a gridded sampling frame is contingent upon the quality of the input settlement layer. The case study showed that the settlement layer enables to detect settlement patterns at high spatial resolution across urban and rural locations. The use of settlement data of lower spatial resolutions would reduce the accuracy of the sampling frame, especially in regions where the built-up area is more scattered. Second, the dimensionality reduction techniques employed to define contextual strata suffer inherent limitations in detecting complex dimensionality structures. Alternative unsupervised classification methods should be tested ^55^. The sampling design can also be affected by the quality of the gridded population data used to define the probability scheme. Even if these gridded data are argued to be more accurate than the related administrative counts ^21^, their fitness for use is contingent upon a number of criteria listed elsewhere ^11^. The use of a nonparametric estimator to assess sampling efficiency also demonstrated systematic oversampling of settled cells with higher population counts when sampling proportional to population size. This involves that larger sample sizes are required within heterogeneous strata.

The proposed grid-sampling design inspired the selection of household survey locations in the Kongo-Central and Kinshasa provinces in 2018 as part of the Geo-Referenced Infrastructure and Demographic Data for Development (GRID3) project. In this project, household survey data collected across small and well-defined geographic areas were used as input data for bottom-up population models to predict basic demographic characteristics across the study area. The survey work conducted as part of this project enabled us to identify critical next steps in the household survey implementation. First, carrying out household surveys within grid cells can be challenging if clear guidelines are not defined in the survey protocol. This includes, for instance, defining the buildings belonging to a cell using the location of their entrance door. The survey work also highlighted other challenges in the implementation of the proposed grid-based sample design related to the difficulty of detecting square grid boundaries in complex settings, as they do not reflect identifiable physical boundaries on the ground (e.g., roads and water bodies). In addition, surveying individual grid cells can be poorly resource-effective in sparsely populated areas. For this reason, a minimum population-count threshold could be enforced by aggregating neighbouring grid cells prior to the sampling design ^10^. This feature has been recently suggested by an automatic enumeration units delineation tool ^56^ and implemented in the latest update of the online version of GridSample, available at https://gridsample.org/.

## Data availability

### Source data

Most of the data used in our case study are freely available and can be accessed following the references presented in Table 1. The official administrative boundaries for the Kongo-Central and Kinshasa provinces are owned by the Central Bureau of the Census (BCR) of the Democratic Republic of the Congo and can be accessed upon reasonable request made to bcrinfo@ins-rdc.org. Further information on the data created by the BCR is available on http://ins-rdc.org.
