# Supplementary material for: A grid-based sample design framework for household surveys
Source: Gates Open Res. 2020 Jan 27;4:13. [Version 1] doi: 10.12688/gatesopenres.13107.1 (PMC7076148; doi:10.12688/gatesopenres.13107.1)
Supplement: Peer review history

# Peer review: Reviewer response for version 1

**DOI:** 10.21956/gatesopenres.14272.r28546

**Published:** 2020-03-12

**Authors:** Tom De Herdt

**Affiliations:** 1 Institute of Development Policy (IOB), University of Antwerp, Antwerp, Belgium

The paper presents an innovative way to provide for an alternative to "bottom-up" sampling for household surveys, by proposing a two-stage "top-down" sampling technique that makes use of all the available geo-datasets -and that duly corrects as much as possible for possible errors.

 I find the exercise generally very convincing and also welcome, especially in cases like the DRC where bottom-up data are virtually absent. 

 I also find the paper particularly well developed, it also very clearly indicates the original data sources, allowing -and almost inviting- readers to engage in further inquiry or replication. 

 The only element I found lacking perhaps is a performance test of the new method compared to the sampling used in one or more existing surveys: would the "newly sampled" results, in the end, significantly differ from the results derived from the usual method? Such an exercise might give a good indication of the value/usefulness of this new sampling method.

I confirm that I have read this submission and believe that I have an appropriate level of expertise to confirm that it is of an acceptable scientific standard.

# Peer review: Reviewer response for version 1

**DOI:** 10.21956/gatesopenres.14272.r28545

**Published:** 2020-03-11

**Authors:** Pierre Goovaerts

**Affiliations:** 1 BioMedware, Inc., Ann Arbor, MI, USA

This well-illustrated paper is proposing a grid-based sample design framework where contextual stratification and proportional to population size sampling are combined to achieve representative sampling for household surveys. This framework is targeted to low- and middle-income countries and is illustrated with case study developed in two provinces located in the western part of the Democratic Republic of Congo.

 I only have a few suggestions to improve the paper:

1. The spatial nature of the data could be incorporated into the classification algorithm using any type of spatially-constrained clustering; either by incorporating a measure of geographical proximity directly into the computation of the dissimilarity matrix (e.g., Oliver and Webster, 1989 ^1^) or the application of contiguity-constrained hierarchical agglomerative clustering approach (e.g., Recchia, 2010 ^2^). This should reduce the salt-and-pepper effect observed by the authors.
2. It might be worth exploring the imposition of a minimum separation distance between sampling units in order to ensure a spatially representative sample while satisfying the other constraints (contextual stratification, proportional to population size sampling).
3. The caption of Fig. 7 should be modified as follows: “the WECDF as coloured lines”.

I confirm that I have read this submission and believe that I have an appropriate level of expertise to confirm that it is of an acceptable scientific standard.

## References

[1] : A geostatistical basis for spatial weighting in multivariate classification. *Mathematical Geology*.1989;21(1) : 10.1007/BF00897238 15-35 10.1007/BF00897238

[2] : Contiguity-Constrained Hierarchical Agglomerative Clustering UsingSAS. *Journal of Statistical Software*.2010;33(Code Snippet 2) : 10.18637/jss.v033.c02 10.18637/jss.v033.c02

# Peer review: Reviewer response for version 1

**DOI:** 10.21956/gatesopenres.14272.r28510

**Published:** 2020-02-04

**Authors:** Alfred Stein

**Affiliations:** 1 Faculty of Geo-information Science and Earth Observation (ITC), University of Twente, Enschede, The Netherlands

A review of ‘A grid-based sample design framework for household surveys’, by Gianluca Boo et al.

 The paper describes the set up and implementation of a household survey carried out in the western Kongo. It is a study of clear interest and relevance, although relatively simple in its different aspects. In fact, the introduction is promising much more than what is delivered in the paper. For instance, the role of geostatistics (hence of spatial dependencies) disappears shortly after equation 1. But what comes out of it in the end, i.e. the implementation, can certainly serve as a ‘framework’. Also the case study has its merits, and in particular figure 7 is convincing. The following changes should be made to make the manuscript acceptable for indexing:

- Adjust the introduction such that it becomes more realistic and in line with the framework as presented.
- The terms ‘frame’ and ‘framework’ need a definition.
- Figure 8, at the left side, has a strange red line, increasing from about 0.18 until 0.35. This artifact of the software should be removed.
- In the discussion section there is a mentioning of representative and non-representative samples. This should be further considered, as so far the sampling is done mainly in a design -based frame. There is literature, notably by Brus et al. that integrate design-based sampling with model-based sampling. I would appreciate it if the authors could add a paragraph on this frame in the discussion section.
- Also: much is *not* considered in this paper, like costs, cost-effectiveness, a justification for the choice of the KS-distance, the role of PCA, and (as often happens in developing countries) extending the sampling to more than one variable. This puts other constraints on the framework. The authors should concentrate on these aspects as well.

I confirm that I have read this submission and believe that I have an appropriate level of expertise to confirm that it is of an acceptable scientific standard, however I have significant reservations, as outlined above.
